# Re-sampling strategy to improve the estimation of number of null hypotheses in FDR control under strong correlation structures

**DOI:** 10.1186/1471-2105-8-157

**Published:** 2007-05-18

**Authors:** Xin Lu, David L Perkins

**Affiliations:** 1 Department of Family and Preventive Medicine, UC San Diego, La Jolla, CA, 92093, USA; 2 Departments of Medicine, UC San Diego, La Jolla, CA, 92093, USA; 3 Departments of Surgery, UC San Diego, La Jolla, CA, 92093, USA

## Abstract

**Background:**

When conducting multiple hypothesis tests, it is important to control the number of false positives, or the False Discovery Rate (FDR). However, there is a tradeoff between controlling FDR and maximizing power. Several methods have been proposed, such as the q-value method, to estimate the proportion of true null hypothesis among the tested hypotheses, and use this estimation in the control of FDR. These methods usually depend on the assumption that the test statistics are independent (or only weakly correlated). However, many types of data, for example microarray data, often contain large scale correlation structures. Our objective was to develop methods to control the FDR while maintaining a greater level of power in highly correlated datasets by improving the estimation of the proportion of null hypotheses.

**Results:**

We showed that when strong correlation exists among the data, which is common in microarray datasets, the estimation of the proportion of null hypotheses could be highly variable resulting in a high level of variation in the FDR. Therefore, we developed a re-sampling strategy to reduce the variation by breaking the correlations between gene expression values, then using a conservative strategy of selecting the upper quartile of the re-sampling estimations to obtain a strong control of FDR.

**Conclusion:**

With simulation studies and perturbations on actual microarray datasets, our method, compared to competing methods such as q-value, generated slightly biased estimates on the proportion of null hypotheses but with lower mean square errors. When selecting genes with controlling the same FDR level, our methods have on average a significantly lower false discovery rate in exchange for a minor reduction in the power.

## Background

Microarray technology has become a standard experimental method in bio-medical research. In the analysis of microarray data, one of the most fundamental tasks is the identification of differentially expressed genes while controlling false positives and minimizing false negatives. This is a multiple hypothesis test problem which analyzes thousands or tens of thousands of genes simultaneously. In these tests we often need to control the false discovery among the rejected hypotheses under a pre-specified level while maintaining maximal power. Thus, there is a trade off in the control of the type-I error between rejecting true null hypotheses (false discovery) versus accepting true alternative hypotheses (false negative).

Traditional Bonferroni correction procedures are designed to control the Family Wise Error Rate (FWER), which guards against making one or more type I errors among a family of hypothesis tests. However, these procedures may be excessively conservative for microarray analysis where the number of hypotheses is very large and a substantial fraction of the genes are differentially expressed [1]. A more appropriate approach is to control the False Discovery Rate (FDR), which is the proportion of type I errors among all rejected hypotheses [2,3]. This approach is particularly useful in exploratory analyses, where the objective is to maximize the discovery of true positives, rather than guarding against one or more false positive results.

A number of methods have been proposed to control the FDR given a population of hypothesis tests. These methods usually assume that the distribution of the test statistics, *f*, can be modeled by a mixture of two components [4]:

*f*(*x*) = *π*_0_*f*_0_(*x*) + (1 - *π*_0_)*f*_1_(*x*)     *π*_0 _= *m*_0_/*m*

Where *f*_0 _is the distribution of the test statistics under H_0_, which by definition equals to 1 when using p-values when tests are independent, *f*_1 _is the distribution of the test statistics under H_1_, *m*_0 _is the number of true H_0_, *m *is the total number of hypotheses under consideration, and *π*_0 _is the proportion of true H_0_. The methods proposed by Benjamini et al [2,3] to control FDR do not estimate *π*_0_; therefore, they provide the strongest controls on FDR but have the lowest power compared to other methods that do so.

In many actual applications where a considerable number of genes are differentially expressed, assuming *π*_0 _= 1 may be too conservative causing loss of power. Several alternative methods, such as nonparametric empirical Bayesian pFDR criterion and its p-value equivalent called q-value method [1,5,6], bin-wise model [7-9], local FDR method [10], parametric beta-uniform mixture models [11-14], the Lowest Slope estimator (LSL) [15], the Spacing LOESS Histogram (SPLOSH) method [16], the nonparametric MLE method [17], the moment generating function approach [18], and the Poisson regression approach [18-20], have all been proposed to estimate *π*_0 _by pooling test statistics and controlling FDR based on the estimated *π*_0_.

In these methods, one of the critical steps is estimating the proportion of null hypotheses, *π*_0_. When using p-values, these estimations usually depend on the assumption that *f*_0 _follows a uniform distribution. This assumption, which is of critical importance for the methods of statistical inference that employ pooling test statistics across genes [21], is valid when all test hypotheses are independent and identically distributed. Furthermore, when there are only weak correlations, or "clumpy" correlations (a large number of groups that have a small number of genes with high correlation within groups but no correlation between groups [21,22]), the uniform assumption is not strongly violated and the method remains adequate. However, in datasets with large scale strong correlations, the joint distribution of the test statistics will no longer be the product of marginal distributions, and the observed *f*_0 _will severely deviate from uniform, causing the current *π*_0 _estimation methods to become very unstable. Increased variation and bias of *π*_0_, as well as FDR, was also observed by Wu et al [14] in datasets with strong local correlations.

The effect of correlation on simultaneous significance tests was previously discussed theoretically [23-25], and a number of permutation based FDR control methods were proposed, such as SAM [26], dChip [27], Ge et al [28], Meinshausen et al [24] and Efron [25]. In these methods, the distribution of *f*_0 _was modeled empirically through permutations, which naturally considered the correlation. However, like Benjamini et al [2,3], these methods don't estimate *π*_0_; therefore, in datasets with a large number of differentially expressed genes, the FDR control may be overly conservative with a loss of power.

Therefore we proposed 2 re-sampling schemes, similar to model averaging in bagging methods, to reduce the variation in estimating *π*_0 _in datasets with strong correlation between gene expression values. Our methods produced a more stable and conservative estimation of *π*_0 _and, therefore, provided stronger control of False Discovery Rate with only a minor sacrifice of power.

## Implementation

### Creating simulated data set

To test the performance of various algorithms in estimating *π*_0_, we generated 2 types of simulated datasets. Both datasets had strong correlation between subsets of genes and a known proportion of true null hypotheses, to represent the log transformed microarray expression data.

#### Data-B

The first simulation method is similar to the method used in Qiu et al [21] and Wu et al [14]. Assume *n *samples and *m *genes, with *n/2 *samples per class. The *m *genes were divided into *K *blocks with each block consisting of *m/K *genes. Assume independence between blocks and constant correlation coefficient between genes within each block. For block *l *and sample *j*, we first created a block center vector

*b*_*lj *_= *d*_*l*_·*x*_*j *_+ *δ*_*lj*_, *l *= 1,...,*K*, *j *= 1,...,*n*

where *d*_*l *_was the mean difference between groups, it equals to *0 *(for simulating non-differentially expressed genes) with probability *π*_0_, or was generated from beta distribution with parameter (4, 20) otherwise; *x*_*j *_was a group indicator; and *δ*_*lj *_was i.i.d. *N(0,1) *to represent sample specific noise. Then the expression value of gene *i *in sample *j *in that block, *Y*_*lji*_, was generated by

*Y*_*lji *_= *ρ*·*b*_*lj *_+ (1 - *ρ*)·*e*_*lji*_, *i *= 1,...,*m*/*K*

where *ρ *was the correlation constant which takes value between [0,1] determining the correlation coefficient between genes within the block, and *e*_*lji *_was i.i.d. *N(0,1)*. The *K *blocks of genes were generated independently of each other and then pooled to form the whole dataset. We call this type of dataset which contains blocks of highly correlated genes Data-B in our experiments.

#### Data-M

The above Data-B model is over-simplified in many aspects, and is still a "clumpy" structure, although the clumpiness can be pronounced. To mimic more realistic situations, we generated a second type of simulated data based on an actual human breast cancer microarray dataset [29] obtained with Affymetrix U133 plus 2.0 microarrays. The dataset contains 65 estrogen receptor positive (ER+) cases and 46 estrogen receptor negative (ER-) cases. The data were normalized by the GCRMA algorithm[30,31], and the gene (probe-set) expression levels were log2-transformed. According to published literatures [32,33], the ER status is one of the most predominant partitioning factors for molecular classification of breast cancer. We therefore took some of the genes differentially expressed between the two classes as "truly H_1_" genes. We selected 8778 genes with differences in mean of log transformed expression levels between the two classes greater than 0.58 (equivalent to a 1.5 fold change). From these genes, each time we randomly chose 1000 to form our simulated dataset and then randomly picked *π*_0 _proportion of the 1000 genes to establish H_0 _genes by scrambling these genes together between samples. Thus, we obtained a simulated dataset with a known number of null hypotheses while the correlations among both the differentially and non-differentially expressed genes were maintained. We call this type of dataset Data-M in our experiments [34].

### Estimating *π*_0 _by re-sampling strategy

To get a better estimation of *π*_0 _in datasets with strong correlations, we proposed 2 re-sampling methods to replace the original *π*_0 _estimation step in q-value method which estimates *π*_0 _directly from the p-value distribution [6].

The first method, termed SampS, re-samples without replacement 2/3 of the samples from each class, calculates p-values for each gene, and then estimates the *π*_0 _from the p-values. For each dataset, we performed this procedure 100 times and used the upper quartile to replace the *π*_0 _estimated by the q-value method.

The second method, termed SampG, re-samples without replacement 2/3 of the values from each class for each gene independently, calculates the p-values for each gene, and then estimates the *π*_0_. We also performed this procedure 100 times for each dataset and used the upper quartile to replace the *π*_0 _estimated by the q-value method.

After each re-sampling step, we have to feed the p-values into a *π*_0 _estimator. This estimator could be the *π*_0 _estimation by q-value method, the mgf method [18], or any other unbiased *π*_0 _estimators. In this paper, we tried to use both q-value and mgf as the plug-in estimator, and called them SampS.q and SampG.q when using q-value as the plug-in estimator, or SampS.m and SampG.m when using mgf as the plug-in estimator.

## Results

### Variation of the *π*_0 _estimation when genes are correlated

**Figure 1:**
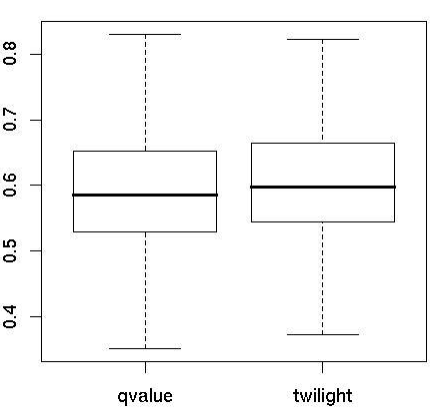
**Estimating the *π*_0 _in an actual microarray data set**. Box plot of *π*_0 _estimated by q-value and twilight methods on 200 bootstrap breast cancer datasets.

To evaluate the impact of correlation structure in large datasets on the estimation of the proportion of true null hypotheses (*π*_0_), we first evaluated current methods on a published microarray dataset [29]. This dataset consists of 65 estrogen receptor positive (ER+) and 46 estrogen receptor negative (ER-) breast cancers. The gene expression levels were normalized by the GCRMA algorithm [30,31] and log2 transformed. Expression values were filtered to eliminate low expressing genes with mean expression below 5 and constant expressing genes with coefficient of variation below 0.1. A total of 9,993 genes passed this filtering criterion. We bootstrapped 200 datasets from this microarray data and used the q-value [6] and twilight [9] methods to estimate the proportion of null hypotheses. The *π*_0 _estimates were similar by the 2 methods on each bootstrapped data set; however, both methods showed a large range of *π*_0 _among the bootstrapped datasets that varied from 0.36 to 0.83 (Figure 1).

**Figure 2:**
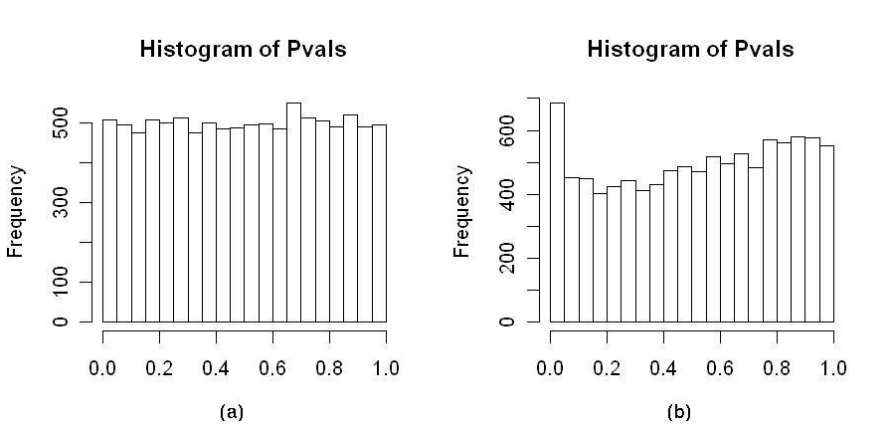
**Histogram of p-values in simulated data sets where all genes were non-differentially expressed**. (a) dataset having 10,000 genes, all independent with each other (b) dataset having 10,000 genes, with 1,000 genes per block.

**Figure 3:**
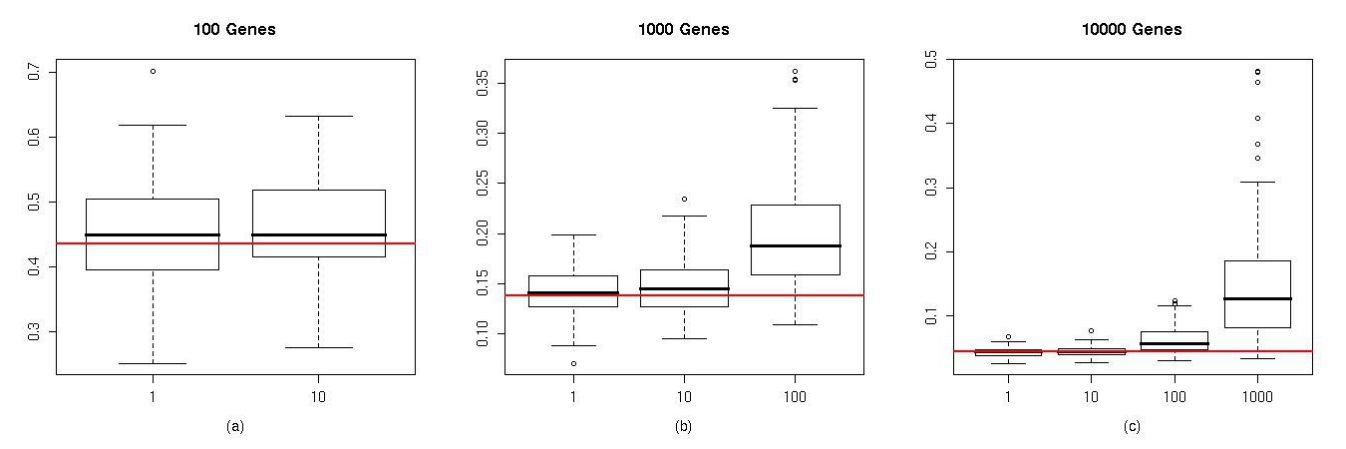
**Box plot of the CV of p-value histogram under different correlation structures**. (a) datasets having 100 genes, with 1 or 10 genes per block. (b) datasets having 1000 genes, with 1, 10 or 100 genes per block. (c) datasets having 10000 genes, with 1, 10, 100 or 1000 genes per block. The histogram was calculated by splitting p-values into 20 bins between [0, 1] with the width of each bin being 0.05. The horizontal lines represent the expected CV when genes are independent.

To further investigate the influence of gene correlation on the estimation of *π*_0_, we generated simulated dataset Data-B which contained blocks of highly correlated genes, but all genes were non-differentially expressed. In our simulation we set the correlation constant *ρ *equal to 0.5, and created datasets containing 100, 1,000 or 10,000 genes with 1, 10, 100 or 1,000 genes per block. After calculating the p-value for each gene, we calculated the coefficient of variation (CV) of the bar heights in the histogram of p-values by splitting the p-values into 20 bins between [0, 1] with the width of each bin being 0.05. Figure 2(a) shows the histogram of p-values in one of the simulations having 10,000 genes all independent with each other, and Figure 2(b) shows the histogram of p-values in another simulation having 10,000 genes with 1,000 genes per block. Comparing Figure 2(a) and 2(b) we can see that with highly correlated genes, the distribution of p-value deviated significantly from uniform, although none of the genes were differentially expressed. We created 100 simulations for each type of data, calculated their CV of the bar height in the p-value histogram, and plotted the box plots of the CVs in Figure 3.

**Figure 4:**
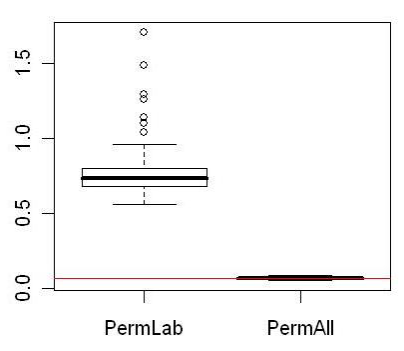
**Box plot of the CV of p-value histogram of permuted microarray datasets**. PermLab: Breast cancer dataset with class labels randomly permuted. By randomly permuting class labels, all genes become non-differentially expressed but the gene-gene correlation intact. PermAll: Randomly permuting all expression values between the 111 samples and 9993 genes. By randomly permuting expression values all genes become non-differentially expressed and independent with each other. The 2 types of permutations were performed 100 times each, and the p-values were split into 20 bins between [0, 1] with the width of each bin being 0.05. The horizontal line represents the expected CV when genes are independent.

In this simulated study, since all genes were non-differentially expressed, the p-values should follow a uniform distribution, and the histogram of p-values should be flat if genes were independent of each other. When the number of correlated genes in each block was small, for example, 1 (independent) or 10 (weak correlation) genes per block, the distribution of p-values approximated a uniform distribution and the CV of the histogram of p-values were close to expected under the independent assumption. However, the CV of the histogram of p-values increased significantly with the growth of correlation structure. In other words, although none of the genes were differentially expressed, the distribution of p-value deviated increasingly from a uniform distribution when large groups of genes were correlated. We also calculated the CV of the histogram of p-values in our microarray dataset by randomly permuting the class labels, which makes all genes non-differentially expressed but still correlated, as well as randomly permuting all expression values across genes and samples which makes genes non-differentially expressed and also independent. The results of 100 permutations showed a dramatically higher CV for the p-value histogram of datasets with only class labels permuted but with gene-gene correlations intact (Figure 4).

Comparing Figure 2, Figure 3 and Figure 4, it is apparent that increased correlation among genes greatly increased the deviation of p-value distribution from uniform. Therefore, strong correlation structures will increase the variation in estimated *π*_0_. And inevitably subsequent statistical inferences and false discovery control procedures would be influenced by this unstable *π*_0 _estimation.

### Improving the *π*_0 _estimation by re-sampling strategies

To improve the estimation of *π*_0 _in datasets with strong correlations, we proposed two methods, termed SampS and SampG, to replace the original *π*_0 _estimation step in the q-value method. In the SampS algorithm, we used a model averaging strategy. We repeatedly sampled 2/3 of the data from each class without replacement, calculated the p-values for genes, estimated *π*_0 _from the p-value distribution and finally used the upper quartile of the re-sampling estimations in the subsequent statistical inferences. In the SampG algorithm, we further broke down the correlations between genes and stabilized the *π*_0 _estimation. In this algorithm, we repeatedly sampled without replacement 2/3 of the expression values from each class independently for each gene, calculated their p-values, estimate *π*_0_, and then used the upper quartile of the re-sampling estimations in subsequent analysis. For the choice of the plug-in *π*_0 _estimator, we tried to use both q-value [6] and moment generating function [18] methods, and called them SampG.q, SampS.q and SampG.m, SampS.m, respectively.

To test the variation in *π*_0 _estimation induced by strong correlation structures, and the performance of our proposed SampS and SampG methods, we created simulated datasets Data-B and Data-M, with true *π*_0 _being 0.9, 0.8, 0.7, 0.6, and the correlation constant *ρ *being 0.3, 0.5 and 0.7, respectively. We created 100 datasets for each combination of these control parameters. In Data-B, we created 1000 genes forming 10 blocks with 100 genes per block to simulate large scale correlation between genes. In Data-M we randomly selected 1000 genes with differences in the mean of log transformed expression levels greater than 0.58, then randomly scrambled 90%, 80%, 70% or 60% of them to create datasets with known proportion of true null hypothesis and strong gene-gene correlations. We applied the SampS and SampG strategies to estimate *π*_0 _for each of the simulated datasets, and compared our results to a number of other methods.

The *π*_0 _estimation methods we used and their corresponding R functions are:

1. The Lowest Slope estimator (LSL) [15] with parameter determined via bootstrap (bootstrap) [5]; function fdr.estimate.eta0

2. q-value method with tuning parameter chosen by smoother method (Qvalue) [6]; function qvalue

3. The Spacing LOESS Histogram (SPLOSH) [16]; function splosh

4. The beta-uniform mixture model (BUM) [12]; function bum.mle

5. The nonparametric MLE method (convest)[17]; function convest

6. The Poisson regression approach (PRE) [18-20]; function p0.mom

7. The moment generating function (mgf) [18]; function p0.mom

8. The Lowest Slope estimator (LSL) [15] with parameter determined adaptively (adaptive) [35]; function fdr.estimate.eta0

9. SampG method, with q-value plug in, 2^nd ^quartile (SampG.q Q2)

10. SampG method, with q-value plug in, 3^rd ^quartile (SampG.q Q3)

11. SampS method, with q-value plug in, 2^nd ^quartile (SampS.q Q2)

12. SampS method, with q-value plug in, 3^rd ^quartile (SampS.q Q3)

13. SampG method, with mgf plug in, 2^nd ^quartile (SampG.m Q2)

14. SampG method, with mgf plug in, 3^rd ^quartile (SampG.m Q3)

15. SampS method, with mgf plug in, 2^nd ^quartile (SampS.m Q2)

16. SampS method, with mgf plug in, 3^rd ^quartile (SampS.m Q3)

**Figure 5:**
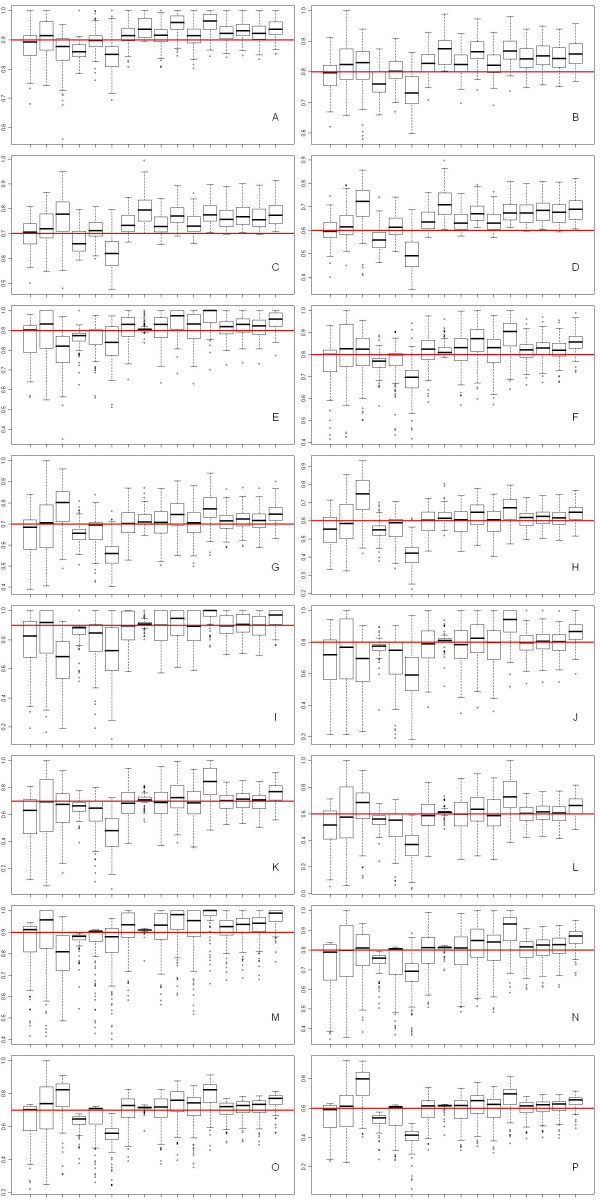
**Boxplot of *π*_0 _estimated by various methods on the simulated data sets**. The methods are (from left to right in each figure): LSL, Qvalue, SPLOSH, BUM, convest, PRE, mgf, adaptive, SampG.q Q2, SampG.q Q3, SampS.q Q2, SampS.q Q3, SampG.m Q2, SampG.m Q3, SampS.m Q2, and SampS.m Q3. A-D: Data-B with *ρ *= 0.3 and *π*_0 _varies from 0.9 to 0.6. E-H: Data-B with *ρ *= 0.5 and *π*_0 _varies from 0.9 to 0.6. I-L: Data-B with *ρ *= 0.7 and *π*_0 _varies from 0.9 to 0.6. M-P: Data-M with *π*_0 _varies from 0.9 to 0.6. Horizontal lines represent the true *π*_0 _in each simulated case.

The *π*_0 _estimations were shown as boxplots in Figure 5, and the Mean Square Error (MSE) were listed in Table 1.

**Table 1:** Mean Square Error of *π*_0 _estimation by different methods

	Data_B												Data_M			
*ρ*	0.3				0.5				0.7							

True *π*_0_	0.9	0.8	0.7	0.6	0.9	0.8	0.7	0.6	0.9	0.8	0.7	0.6	0.9	0.8	0.7	0.6

Bootstrap	0.0038	0.0029	0.0035	0.0030	0.0106	0.0113	0.0115	0.0105	0.0423	0.0495	0.0422	0.0336	0.0202	0.0227	0.0213	0.0164
Qvalue	0.0045	0.0054	0.0051	0.0049	0.0120	0.0190	0.0170	0.0139	0.0445	0.0592	0.0621	0.0622	0.0247	0.0309	0.0318	0.0214
SPLOSH	0.0061	0.0059	0.0119	0.0201	0.0233	0.0107	0.0173	0.0308	0.0909	0.0456	0.0279	0.0298	0.0239	0.0145	0.0225	0.0388
BUM	0.0040	0.0032	0.0032	0.0040	0.0042	0.0037	0.0045	0.0061	0.0097	0.0050	0.0060	0.0060	0.0041	0.0071	0.0090	0.0117
Convest	0.0018	0.0021	0.0024	0.0021	0.0076	0.0087	0.0079	0.0076	0.0367	0.0402	0.0339	0.0275	0.0177	0.0200	0.0173	0.0138
PRE	0.0063	0.0072	0.0103	0.0151	0.0135	0.0189	0.0249	0.0402	0.0690	0.0739	0.0746	0.0748	0.0225	0.0289	0.0343	0.0478
Mgf	0.0017	0.0026	0.0038	0.0039	0.0054	0.0060	0.0048	0.0043	0.0122	0.0152	0.0148	0.0130	0.0104	0.0114	0.0094	0.0076
Adaptive	0.0030	0.0075	0.0144	0.0175	0.0008	0.0017	0.0023	0.0028	0.0012	0.0024	0.0025	0.0019	0.0003	0.0005	0.0005	0.0010
SampG.q Q2	0.0017	0.0026	0.0035	0.0032	0.0059	0.0067	0.0054	0.0049	0.0135	0.0168	0.0162	0.0140	0.0114	0.0124	0.0103	0.0085
SampG.q Q3	0.0043	0.0069	0.0084	0.0077	0.0065	0.0100	0.0076	0.0062	0.0106	0.0163	0.0176	0.0160	0.0109	0.0137	0.0119	0.0091
SampS.q Q2	0.0017	0.0026	0.0036	0.0033	0.0062	0.0070	0.0055	0.0051	0.0138	0.0176	0.0169	0.0169	0.0138	0.0136	0.0122	0.0094
SampS.q Q3	0.0046	0.0072	0.0091	0.0083	0.0073	0.0139	0.0110	0.0091	0.0081	0.0233	0.0341	0.0384	0.0094	0.0187	0.0176	0.0122
SampG.m Q2	0.0016	0.0037	0.0067	0.0082	0.0027	0.0029	0.0027	0.0025	0.0058	0.0060	0.0057	0.0047	0.0046	0.0045	0.0037	0.0032
SampG.m Q3	0.0021	0.0046	0.0081	0.0097	0.0030	0.0033	0.0031	0.0027	0.0055	0.0060	0.0057	0.0048	0.0048	0.0046	0.0039	0.0032
SampS.m Q2	0.0017	0.0037	0.0068	0.0083	0.0028	0.0031	0.0030	0.0027	0.0062	0.0063	0.0060	0.0050	0.0051	0.0048	0.0042	0.0033
SampS.m Q3	0.0025	0.0053	0.0091	0.0109	0.0045	0.0053	0.0049	0.0044	0.0059	0.0098	0.0097	0.0087	0.0065	0.0063	0.0056	0.0037

Table 1 listed the MSE of *π*_0 _estimations by various methods. To better understand the methods tested, we listed both the 2^nd ^and 3^rd ^quartiles of the SampS and SampG methods compared to other methods. Later, we used only the 3^rd ^quartile to provide strong control of FDR. From Figure 5 and Table 1 we can see that the bootstrap, Qvalue and SPLOSH methods are very sensitive to correlation and have higher MSE, especially the SPLOSH methods tend to under-estimate higher *π*_0 _but over-estimate lower *π*_0_; the BUM, convest and PRE methods tend to under-estimate *π*_0 _in most of the simulated data sets with strong correlations, which is not favorable in FDR controls; the adaptive method tends to over-estimate *π*_0 _when the correlation is not very strong, which is also observed by [18] with independent and weak correlated data, but it worked better on cases with strong correlations when the MSE were the least among all tested methods; mgf method is generally the second best among the above, with relatively small bias and variation among all simulated cases. In terms of SampS and SampG methods, both the 2^nd ^and 3^rd ^quartile outperformed the corresponding plug-in estimator, due to smoothed variation by model averaging, and SampG performs better than SampS with smaller MSE because SampG breaks down correlation between genes whereas SampS does not. And since mgf outperforms the q-value method, the SampG and SampS with the mgf plug-in also outperforms SampG and SampS with the q-value plug-in.

**Figure 6:**
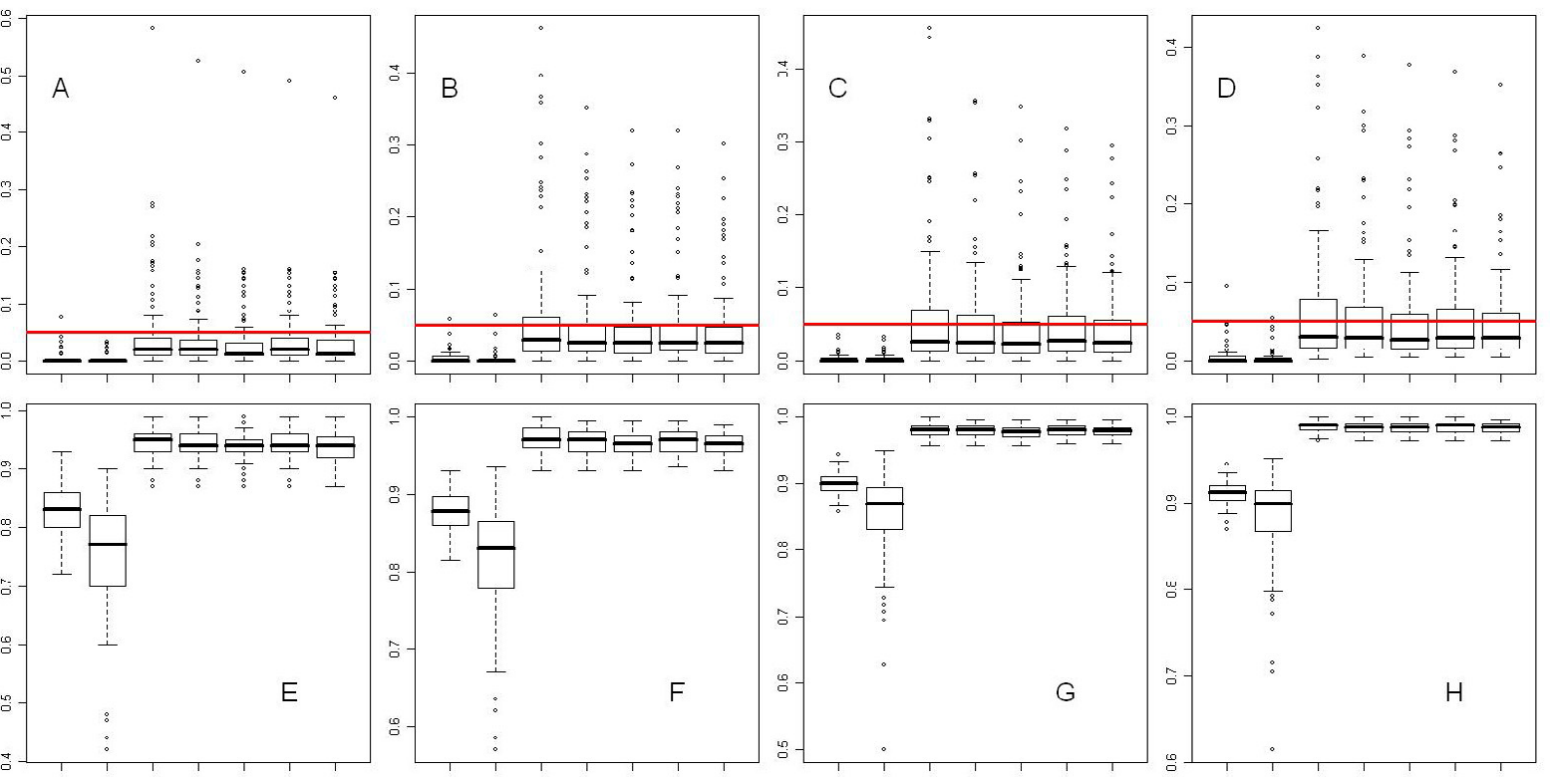
**Boxplot of actual FDR and power by various methods on Data-M**. The methods are (from left to right in each figure): BY, howmany, Qvalue, SampG.q, SampS.q, SampG.m, SampS.m. A-D: boxplot of FDR on Data-M with *π*_0 _varies from 0.9 to 0.6. E-H: boxplot of power on Data-M with *π*_0 _varies from 0.9 to 0.6. Horizontal lines represent FDR at 0.05 level.

We then selected genes with FDR controlled under 0.05 level based on the *π*_0 _estimated by our method, calculated the actual false discovery rate and power, and compared our results to that of FDR controlling method proposed by Benjamini et al. with correlations considered (BY; function p.adjust with method "BY") [3], the permutation based FDR controlling method (howmany; function howmany_dependent) [24], and q-value method [6]. The boxplot of actual false discovery rate and power on the 4 types of Data-M simulations were shown in Figure 6. From Figure 6 it can be seen that both the BY and howmany methods provided strong control of FDR, their actual FDR level was much lower than 0.05, and their power to detect true alternatives were much lower than methods where the proportion of true null hypotheses was estimated and used in the FDR control. Comparing the q-value method and our proposed SampS and SampG method, for majority of the cases the actual FDR level were still controlled below 0.05 level, although some outliers exist with actual FDR up to 0.4~0.6, due to the unstable pi_0 estimations. The SampS and SampG methods, especially when using mgf as the plug-in *π*_0 _estimator, tend to have lower FDR on those outlier cases compared to q-value method. The differences in power between q-value method and our proposed methods were very minor, compared to the difference between q-value and BY method. We also tested on the Data-B simulations, and obtained the same result. Since we used the 3^rd ^quartile of *π*_0 _estimated by SampS and SampG, our estimations were biased but conservative. With the same FDR control level our method would make smaller numbers of rejections than the q-value method, therefore the actual FDR and power of the genes selected were lower than that of the q-value method. This was shown by the p-value in pair-wise comparison of actual FDR and power between the re-sampling based methods and q-value method in these simulations, where all FDR and most power comparisons were significant (Table 2, Table 3). Interestingly, comparing the mean of FDR and power, as shown in Table 4 and 5, we found that the SampS and SampG methods, compared to the q-value method, can reduce the average FDR up to 40%, with a decrease of average power in most cases less than 1%. In fact, with the highest correlation constant we tested, and in most cases for SampG.m method, the decrease of power was not even significant from the q-value method. In contrast, the most conservative BY method reduced the average FDR by more than 90% compared to the q-value method, but also reduced the average power by approximately 10% in cases with strong correlation.

**Table 2:** P-values comparing FDR by different methods.

	*ρ*	True *π*_0_	BY	SampG.q	SampS.q	SampG.m	SampS.m
Data-B	0.3	0.9	3.25E-11	3.68E-01	1.56E-02	1.31E-01	1.29E-01
		0.8	7.45E-26	8.70E-04	8.40E-04	7.20E-03	4.12E-03
		0.7	2.80E-32	6.23E-07	1.34E-07	4.13E-06	2.38E-06
		0.6	9.74E-46	6.00E-09	6.10E-08	1.92E-08	4.47E-09
	0.5	0.9	1.28E-10	1.74E-03	3.68E-05	4.56E-03	6.45E-04
		0.8	4.17E-14	8.92E-06	5.09E-06	1.81E-04	2.94E-05
		0.7	3.12E-21	4.94E-08	5.46E-10	1.40E-05	2.12E-07
		0.6	2.19E-21	1.36E-07	2.81E-10	1.20E-06	1.75E-08
	0.7	0.9	4.20E-06	1.26E-02	1.11E-03	2.86E-02	6.62E-03
		0.8	1.32E-07	2.00E-04	2.62E-05	3.48E-04	1.15E-04
		0.7	5.96E-10	2.12E-03	1.49E-04	1.62E-03	4.58E-04
		0.6	4.06E-10	6.75E-04	3.24E-05	6.96E-04	1.52E-04
Data-M		0.9	4.92E-08	8.63E-05	1.97E-05	4.39E-04	4.16E-05
		0.8	1.01E-09	3.05E-05	5.13E-07	4.84E-05	7.05E-06
		0.7	2.76E-10	3.31E-05	1.83E-06	1.70E-04	2.28E-05
		0.6	1.61E-12	8.38E-07	5.29E-09	6.91E-06	6.11E-07

The actual FDR in gene selections across the 100 simulations under each configuration by these methods was compared to that of q-value method using one-tail paired t-statistics.

**Table 3:** P-values comparing power by different methods.

	*ρ*	True *π*_0_	BY	SampG.q	SampS.q	SampG.m	SampS.m
Data-B	0.3	0.9	3.71E-17	1.10E-04	1.06E-04	2.06E-02	8.37E-03
		0.8	2.74E-31	3.93E-05	2.97E-05	1.94E-03	5.58E-04
		0.7	7.82E-34	7.72E-08	2.57E-08	3.20E-07	1.09E-07
		0.6	4.48E-48	8.31E-12	9.01E-12	2.78E-12	5.55E-13
	0.5	0.9	1.67E-08	4.95E-03	3.35E-03	3.73E-02	7.94E-03
		0.8	1.06E-13	7.70E-04	6.05E-06	4.06E-02	9.28E-04
		0.7	6.42E-15	2.14E-03	1.12E-04	1.60E-02	2.37E-03
		0.6	1.28E-17	5.55E-04	1.77E-06	2.99E-03	1.41E-04
	0.7	0.9	9.89E-03	8.65E-02	4.81E-02	6.76E-02	5.24E-02
		0.8	4.72E-06	2.38E-02	1.11E-02	2.89E-02	1.73E-02
		0.7	2.65E-08	1.53E-01	7.25E-03	2.85E-01	5.92E-02
		0.6	1.73E-06	1.19E-01	2.52E-02	1.25E-01	5.93E-02
Data-M		0.9	1.77E-53	9.78E-05	1.00E-06	3.66E-03	1.37E-05
		0.8	1.69E-62	3.05E-06	2.16E-10	3.52E-04	7.14E-08
		0.7	1.53E-74	5.27E-03	4.66E-08	3.86E-01	6.87E-03
		0.6	2.37E-80	2.05E-04	3.42E-10	2.49E-01	1.34E-05

The actual power in gene selections across the 100 simulations under each configuration by these methods was compared to that of q-value method using one-tail paired t-statistics.

**Table 4:** Percentage in decrease of mean FDR

	*ρ*	True *π*_0_	BY^a^	SampG.q^b^	SampS.q^c^	SampG.m^d^	SampS.m^e^
Data-B	0.3	0.9	84.13%	2.19%	3.00%	1.99%	2.05%
		0.8	90.03%	5.54%	5.60%	4.04%	4.33%
		0.7	87.04%	7.64%	8.27%	7.44%	7.84%
		0.6	91.59%	6.82%	6.72%	7.09%	7.45%
	0.5	0.9	92.89%	4.51%	8.51%	1.83%	6.39%
		0.8	92.87%	7.68%	9.73%	3.90%	7.66%
		0.7	95.09%	6.79%	11.63%	3.01%	8.24%
		0.6	94.24%	8.49%	13.26%	4.34%	8.82%
	0.7	0.9	96.62%	19.79%	35.22%	18.30%	25.52%
		0.8	97.03%	28.32%	40.86%	26.00%	32.72%
		0.7	96.15%	17.09%	35.83%	14.98%	23.57%
		0.6	97.80%	20.12%	36.47%	15.41%	27.46%
Data-M		0.9	94.87%	10.25%	14.66%	7.00%	14.70%
		0.8	93.11%	7.87%	19.48%	6.33%	11.38%
		0.7	93.50%	4.60%	12.11%	0.15%	5.96%
		0.6	94.18%	6.35%	14.16%	1.85%	8.70%

^a ^(FDR_qvalue _- FDR_BY_)/FDR_qvalue_

^b ^(FDR_qvalue _- FDR_SampG.q_)/FDR_qvalue_

^c ^(FDR_qvalue _- FDR_SampS.q_)/FDR_qvalue_

^d ^(FDR_qvalue _- FDR_SampG.m_)/FDR_qvalue_

^e ^(FDR_qvalue _- FDR_SampS.m_)/FDR_qvalue_

**Table 5:** Percentage in decrease of mean power

	*ρ*	True *π*_0_	BY^a^	SampG.q^b^	SampS.q^c^	SampG.m^d^	SampS.m^e^
Data-B	0.3	0.9	48.57%	4.45%	3.93%	3.38%	3.54%
		0.8	41.43%	1.96%	2.06%	1.58%	1.79%
		0.7	36.81%	1.89%	2.01%	2.30%	2.42%
		0.6	34.73%	1.58%	1.63%	2.00%	2.18%
	0.5	0.9	18.21%	0.47%	0.52%	0.40%	0.51%
		0.8	11.02%	0.28%	0.46%	0.16%	0.34%
		0.7	10.55%	0.26%	0.36%	0.21%	0.28%
		0.6	10.38%	0.33%	0.57%	0.36%	0.53%
	0.7	0.9	5.67%	0.34%	0.52%	0.38%	0.54%
		0.8	5.73%	0.83%	1.21%	0.98%	1.15%
		0.7	7.91%	0.14%	0.49%	0.09%	0.31%
		0.6	4.40%	0.16%	0.41%	0.21%	0.35%
Data-M		0.9	11.85%	0.36%	0.53%	0.27%	0.53%
		0.8	9.50%	0.19%	0.36%	0.17%	0.36%
		0.7	7.99%	0.09%	0.23%	0.01%	0.12%
		0.6	7.76%	0.08%	0.19%	0.02%	0.15%

^a ^(Power_qvalue _- Power_BY_)/Power_qvalue_

^b ^(Power_qvalue _- Power_SampG.q_)/Power_qvalue_

^c ^(Power_qvalue _- Power_SampS.q_)/Power_qvalue_

^e ^(Power_qvalue _- Power_SampG.m_)/Power_qvalue_

^f ^(Power_qvalue _- Power_SampS.m_)/Power_qvalue_

### Comparing the re-sampling strategy to bootstrap p-values directly

When there is strong correlation between genes, and thus also between p-values, bootstrapping p-values does not change the correlation structure and therefore the estimations are still unstable. In contrast, re-sampling of samples or gene expression values as we proposed could address the variations induced by the correlation structure and therefore smooth the estimation.

**Figure 7:**
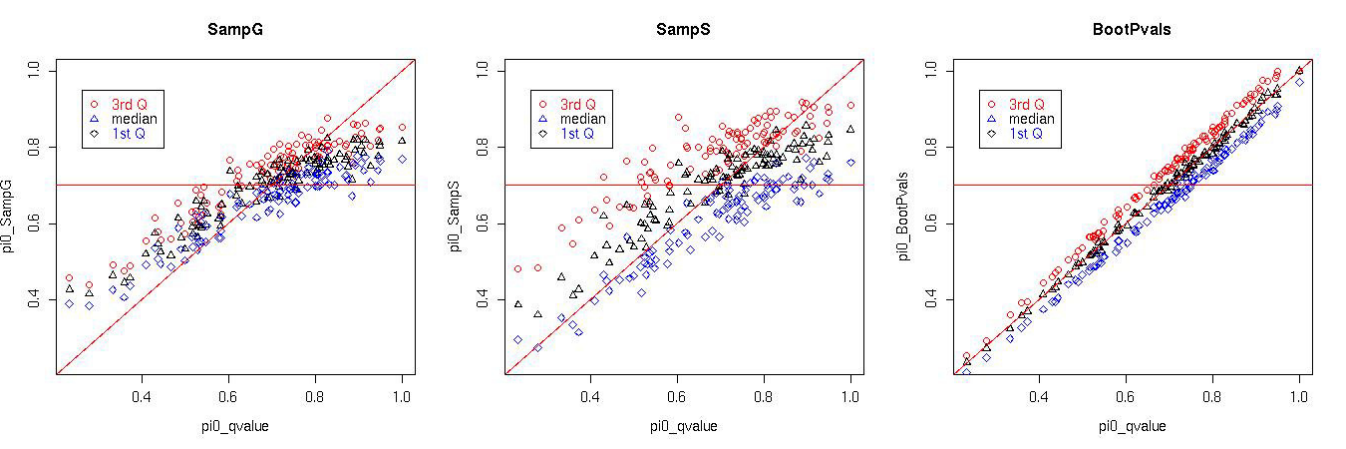
**Scatter plot of *π*_0 _estimated by q-value method versus the 3 quartiles by SampG, SampS and bootstrapping p-values**. X-axis represents the *π*_0 _estimated by q-value method on the 100 Data-M simulations, and y-axis represents the 1^st^, 2^nd ^and 3^rd ^quartiles of *π*_0 _estimated by SampG, SampS method and the bootstrapped p-values, in the 3 plots respectively. Circles are the 3^rd ^quartile of estimation, triangles are the median and diamonds are the 1^st ^quartile. The lines in the images are the 45 degree diagonal line and the horizontal line corresponds to the true *π*_0 _which equals to 0.7. We used q-value plug-in for SampG and SampS in this figure.

For example, for each of the simulated datasets, we bootstrapped the p-values 100 times and then estimated *π*_0 _by the q-value method. Figure 7 shows the scatter plots of *π*_0 _estimated by the q-value method versus the 1^st^, 2^nd ^and 3^rd ^quartiles of the SampS and SampG methods, as well as the estimations by bootstrapping p-values on the 100 Data-M simulations with true *π*_0 _equals to 0.7. For the bootstrap method, as expected, the scatter plot showed that the median estimation of *π*_0 _was close to the estimation using original p-values for all cases. Whereas for the SampS and SampG methods, since they smoothed the variation induced by correlation between p-values, the variation was much smaller than the q-value method. Especially for cases where the q-value method severely under- or over-estimated the *π*_0_, the estimation by the SampS and SampG methods were closer to the true value. This was also observed in all simulated datasets that we have tested (data not shown).

## Discussion

In actual microarray datasets, genes expression is often correlated due to co-regulation, sharing of transcription factor binding motifs, or technical reasons such as sequence similarity, cross-hybridization or signal leak during hybridization. This is of critical importance for statistical inferences that rely on pooling of test statistics across genes [21]. The distribution of p-values of these correlated genes can be viewed under a mixture model where groups of highly correlated genes share similar p-values and the whole distribution is actually a mixture of components corresponding to the groups of highly correlated genes. The effect of strong and large scale correlations is equivalent to reducing the total number of independent components in this mixture model. Storey [22] argued that subsets of genes fall into small but highly correlated groups due to co-regulation or cross-hybridization, but these groups are small in size and nearly independent with each other ("clumpy dependency"), therefore the uniformity of p-value distribution of true null genes would not be strongly affected. However, other researchers, such as Qiu et al [21], have found that the impact of correlation may be quite strong. It is also true that in our permutation study on a breast cancer microarray dataset, the distribution of p-values could be extremely far from uniform due to gene-gene correlation.

Systems biology research has shown that biological gene networks have a scale free [36], hierarchical structure [37,38], where most of the genes are connected to a small number of other genes forming small groups of complexes, while some "hub" genes may be connected to large number of peripheral genes. The distribution of connectivity degree (the number of genes being connected to a given gene) decreases with a power-law, which is much slower than the exponential decay expected in a random network [36]. These gene-gene interactions may be reflected by co-regulation or correlation in expression under certain conditions, and the possible scale of gene interaction is unlimited given the scale free structure. Therefore, large scale correlation of gene expression levels is not surprising in microarray studies.

We have shown in our simulated study that with the growth of correlation structures, the p-value distribution of H_0 _genes increasingly deviates from a typical uniform distribution. This may influence the estimation of *π*_0 _and the following statistical inferences. The effect of strong correlation was also discussed by other researchers [14]. To solve this problem, we proposed the SampS and SampG methods. These algorithms replaced the unbiased but unstable *π*_0 _estimation step in the q-value method with a model averaging procedure of re-sampling samples or furthermore re-sampling independently for each gene to partially break the correlations between genes. Strong correlation between genes will inevitably increase the variation of the *π*_0 _estimation, even though the variation could be partially smoothed by the re-sampling strategies proposed in this paper. Therefore, it is necessary to compromise between safety and efficiency; in this case, we would like to shrink the estimation toward 1 from an unbiased estimation to guarantee a strong control of FDR. That is why we used the upper quartile instead of the median of the re-sampling estimations, although medians had a smaller MSE in estimating *π*_0 _in our simulated studies. We showed in our simulations that these plug-in revisions, compared to the q-value method, can greatly reduce the variation of the *π*_0 _estimation under strong gene-gene correlations, and enhance the performance of FDR control by reducing false discovery rate up to 40% with a reduction of power less than 1% compared to q-value method.

In our study, to create datasets with a known proportion of true null hypotheses while still having a similar correlation structure to that in actual microarray datasets, we developed 2 strategies to generate simulated datasets. The first one, Data-B, is simply a block-wise structure with arbitrary block size and intra-block correlation, but independent between blocks. When the block size was small, this was similar to the "clumpy" hypothesis [22]. When the block size became bigger, we showed that the correlations influenced the *π*_0 _estimation, and the re-sampling strategies we proposed improved the performance of gene selection by significantly reducing the FDR with a minor reduction of power. To mimic a more realistic scenario, we also developed the Data-M strategy to generate simulated data from actual microarray datasets by arbitrarily permuting a given proportion of the genes. This permutation breaks the correlation between arbitrarily assigned differentially and non-differentially expressed genes, but maintains the correlation within the 2 groups of genes.

## Conclusion

The SampS and SampG methods we proposed and tested in this paper are simple revisions, but they greatly improved the *π*_0 _estimations. The same approach using independent re-samples of expression values to estimate the *π*_0 _and then using the upper quartile of the re-sampling estimations in FDR control, could be applied to other FDR algorithms in data where strong correlation between hypotheses exists. In this paper we considered the typical 2-sample comparison problem with a reasonable number of independent replicates. For more complex problems, such as time-course studies, the SampG method (re-sample gene expression values independently for each gene) may not be able to be applied directly without a reasonable number of replicates in each time point, but the SampS method (re-sample samples) may be applicable if there is sound reason to assume the existence of stationary time patterns in the biological system under investigation.

## Availability and requirements

The R code of SampG and SampS methods, and R code to generate simulated data sets Data-B and Data-M, is included as Additional file 1.

Requirements: R environment.

Operating systems: Windows XP or Linux.

License: free.

## Authors' contributions

XL developed the basic strategies of SampG and SampS methods, developed the methods to generate Data-B and Data-M simulation data, and did all simulation studies. DPL helped XL on analyzing the experiments, comparing the methods, and improving the algorithms. XL and DPL executed the writing together.

## Supplementary Material

Additional file 1 The file contains the R source code of functions to create Data-B, Data-M simulated data sets, and SampG, SampS methods to estimate the *π*_0_. Click here for file
